# Modifiable lifestyle behaviors, but not a genetic risk score, associate with metabolic syndrome in evening chronotypes

**DOI:** 10.1038/s41598-017-18268-z

**Published:** 2018-01-17

**Authors:** Beatriz Vera, Hassan S. Dashti, Purificación Gómez-Abellán, Antonio M. Hernández-Martínez, Alberto Esteban, Frank A. J. L. Scheer, Richa Saxena, Marta Garaulet

**Affiliations:** 1 Department of Physiology, University of Murcia, Murcia, Spain; 2 IMIB-Arrixaca, Murcia, Spain; 3 Center for Genomic Medicine, Massachusetts General Hospital, Boston, Massachusetts, USA; 4 Department of Endocrinology and Nutrition, “Virgen Arrixaca” Hospital and University of Murcia, Murcia, Spain; 5 Garaulet Nutrition Centers, Murcia, Spain; 6 Division of Sleep and Circadian Disorders, Brigham and Women’s Hospital, Boston, Massachusetts, USA; 7 Division of Sleep Medicine, Harvard Medical School, Boston, Massachusetts, USA

## Abstract

Evening chronotype associates with health complications possibly via lifestyle factors, while the contribution of genetics is unknown. The aim was to study the relative contributions of genetics, lifestyle, and circadian-related physiological characteristics in metabolic risk of evening chronotype. In order to capture a biological contribution to chronotype, a genetic-risk-score (GRS), comprised of 15 chronotype-related variants, was tested. Moreover, a wide range of behavioral and emotional eating factors was studied within the same population. Chronotype, lifestyle, and metabolic syndrome (MetS) outcomes were assessed (*n* = 2,126), in addition to genetics (*n* = 1,693) and rest-activity/wrist-temperature rhythms (*n* = 100). Evening chronotype associated with MetS and insulin resistance (*P* < 0.05), and several lifestyle factors including poorer eating behaviors, lower physical activity and later sleep and wake times. We observed an association between higher evening GRS and evening chronotype (*P* < 0.05), but not with MetS. We propose a GRS as a tool to capture the biological component of the inter-individual differences in chronotype. Our data show that several modifiable factors such as sedentary lifestyle, difficulties in controlling the amount of food eaten, alcohol intake and later wake and bed times that characterized evening-types, may underlie chronotype-MetS relationship. Our findings provide insights into the development of strategies, particularly for evening chronotype.

## Introduction

Chronotype is a characteristic that determines an individual’s circadian preference^1,2^. This diurnal preference allows people to be classified as morning (i.e., early) or evening (i.e., late) chronotypes^3^. Chronotype preferences are in part determined by the timing of endogenous circadian rhythms. Morning and evening chronotypes may be shifted by 2 to 3 hours in the timing of the rhythms in body temperature, melatonin, cortisol, and other hormone secretions^4^. In general, more evening chronotype has been observed to be associated with health complications such as obesity-related metabolic alterations, negative psychological outcomes, including depressive and anxiety symptoms, and poor glycemic control^5^.

Genetics may be implicated in the connection between evening chronotype and health complications. Results obtained from classical twins studies have estimated that genetic factors are responsible for 46% to 70% of variance in the heritability of daily rhythms^6^. More recently, three genome-wide association studies performed in participants of European descent from the UK Biobank [*n* = 100,420^7^; and *n* = 128,266^8^] and from databases of the US genetics company, 23andMe, [*n* = 89,283^9^] have observed several single nucleotide polymorphisms (SNPs) associating with chronotype.

Lifestyle factors may in part explain the associations between evening chronotype and the observed metabolic alterations. In terms of feeding behavior, evening chronotype has been associated with suboptimal dietary habits, such as larger caloric intake in the evenings^10^ and fewer servings of fruits and vegetables^11^, and lower dietary restraint^12^. People with an evening chronotype also have later times of behaviors including later meal intake^10^, sleep onset^13^, and physical activity^14^. In addition, evening chronotype individuals are more likely to suffer from chronic sleep curtailment as a result of later bed times at night and early wake time due to social demands^15^. These lifestyle factors have independently been linked to various health complications^16^.

Previous attempts to understand the relationship between chronotype and metabolic disturbances have been limited in their scope. The link between chronotype and metabolic disorders has been proposed to be mediated by changes in lifestyle, and the contribution of genetics to this connection has not been explored yet. Based on findings from recent chronotype GWAS studies, we have developed a genetic risk score (GRS) of 15 common chronotype genetic variants in order to investigate whether genetics, representing a biological component, may play a role in inter-individual differences in chronotype and in features of metabolic disorder. Furthermore, most lifestyle factors related to chronotype have been studied separately in independent populations: some are focused primarily on physical activity habits^17^, others on sleep^18^, and very few have considered a wider range of behavioral and emotional eating factors, particularly within the same population^19^. Thus, the aim of the current study was to study the relative contributions of genetics, lifestyle, and circadian-related physiological characteristics in metabolic risk of evening chronotype. In order to capture the biological contribution to chronotype, a GRS comprised of 15 chronotype-related variants, was tested. Moreover, a wide range of behavioral and emotional eating factors was studied within the same population.

## Results

### Chronotype and Metabolic Syndrome

The present population of 2,126 participants was comprised of 1,110 (52%) more morning chronotype and 1,016 (48%) more evening chronotype (median ME score = 53). Evening types showed greater adverse metabolic outcomes in components of the metabolic syndrome including higher BMI, higher triglycerides, lower HDL-cholesterol, a higher HOMA-IR and a significantly higher total MetS Score (p < 0.05; Table 1). The association between chronotype and MetS remained significant even after accounting for GRS for chronotype in the model (p = 0.042).

**Table 1:** General characteristics of ONTIME population.

	Morning Chronotype (Mean ± SEM) *n* = 1,110	Evening Chronotype (Mean ± SEM) *n* = 1,016	*P-value*	*P-trend*
Age (y)	42.97 ± 12.67^a^	36.17 ± 12.68^a^	<**0.001**	<**0.001**
%Women, n (%)	902 (81.3)	820 (80.7)	0.746	—
BMI (kg/m^2^)	30.99 ± 0.16	31.31 ± 0.16	0.157	**0.032**
Body fat (%)	37.03 ± 0.19	36.98 ± 0.19	0.849	0.543
***Metabolic syndrome and insulin resistance***				
Waist (cm)	102.21 ± 0.40	102.52 ± 0.42	0.592	0.236
Fasting glucose (mg/dl)	85.09 ± 0.45	85.34 ± 0.48	0.714	0.738
Triglycerides (mg/dl)*	100.63 ± 1.71	105.00 ± 1.79	**0.009**	**0.006**
HDL-c (mg/dl)	57.07 ± 0.46	55.57 ± 0.48	**0.026**	**0.001**
Systolic Pressure (mmHg)	11.63 ± 0.04	11.64 ± 0.05	0.901	0.268
Diastolic pressure (mmHg)	7.25 ± 0.03	7.22 ± 0.03	0.563	0.650
MetS Score*	2.06 ± 0.04	2.16 ± 0.04	**0.011**	**0.050**
Insulin (µUI/ml)*	7.40 ± 0.22	7.62 ± 0.23	0.112	<**0.001**
HOMA-IR*	1.61 ± 0.05	1.68 ± 0.06	0.360	**0.002**

Chronotype was dichotomized into “more morning” (Morning type) and “more evening” (Evening type) based on the median ME score of the total population (<53, more evening; ≥53, more morning).

Abbreviations: BMI, Body mass index; MetS, Metabolic Syndrome; HDL-C, High Density Lipoprotein – Cholesterol; VLDL-C, Very Low Density Lipoprotein-Cholesterol; HOMA-IR, Homeostatic Model Assessment – Insulin Resistance.

Adjusted by sex, age, clinic site and number study.

^a^M ± SD.

*Values were logarithmically transformed.

*P*-value refers to association between morning and evening chronotype and exposures of interest. *P*-trend refers to the continuous association between the ME score and exposures of interest.

### Chronotype and Genetic risk score

**Figure 1:**
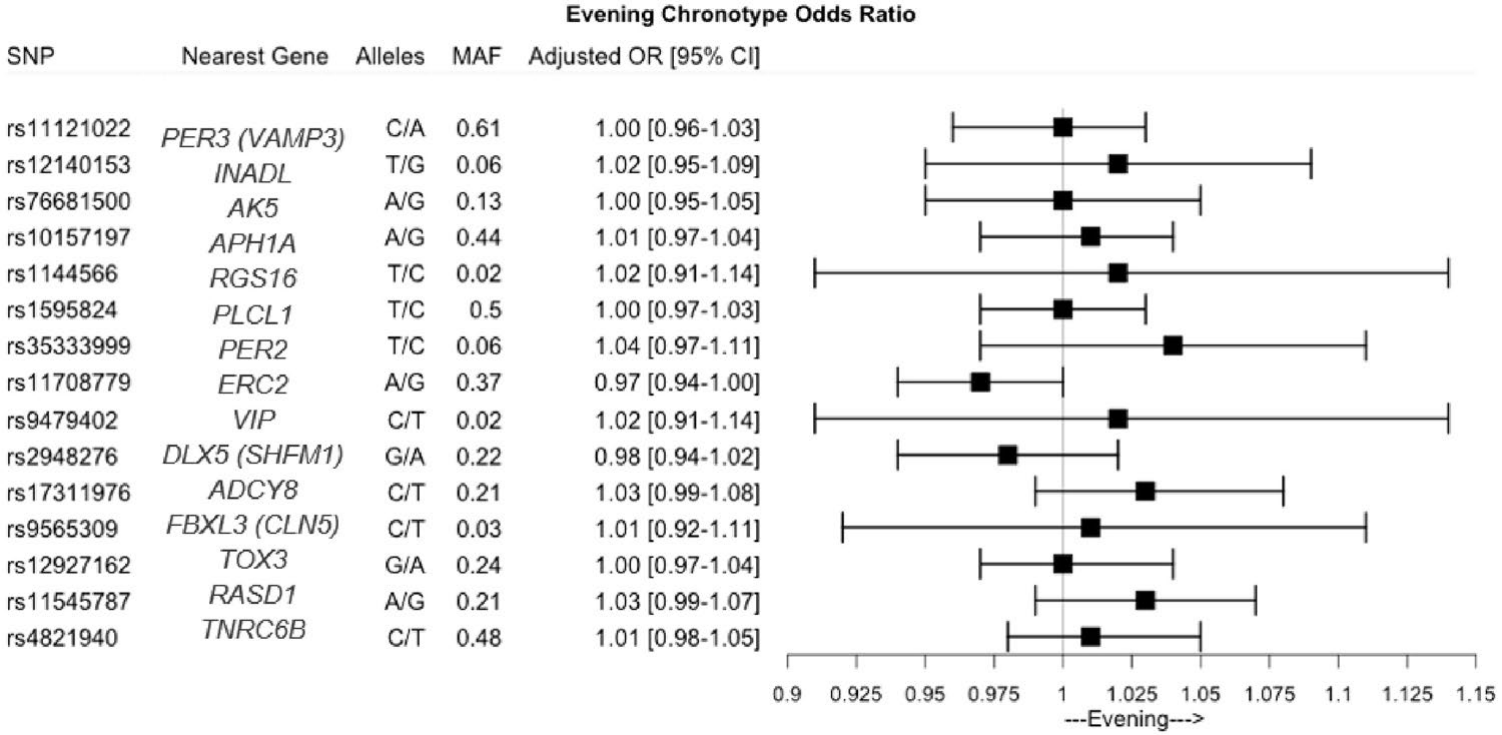
Odds ratio and 95% CI of the individual chronotype SNPs associations with self-reported evening chronotype (ME Score >53) in ONTIME population.

Our GRS of 15 chronotype genetic variants was able to capture chronotype in the current population, with a higher evening GRS associated with more evening chronotype (Fig. 1, Table 2, Supplemental Table 1). Despite associations with more evening chronotype, we observed that the GRS did not associate with metabolic syndrome and insulin resistance (Table 2). Interestingly, the GRS was associated with systolic blood pressure such that a more evening GRS was associated with higher blood pressure whereas each additional risk allele is associated with 0.054 mmHg (SE = 0.027) higher systolic blood pressure (*P* = 0.043).

**Table 2:** Genetic Risk Score (GRS) association with chronotype, food timing and metabolic syndrome components (*n* = 1,693).

	GRS Score	
	*B* (SE)	*P*
***Chronotype***		
ME Score	−0.121 (0.058)	**0.037**
***Metabolic syndrome and insulin resistance***		
MetS Score*	0.001 (0.001)	0.52
Fasting glucose (mg/dl)	−0.150 (0.160)	0.35
Triglycerides (mg/dl)*	−0.003 (0.003)	0.41
HDL-c (mg/dl)	−0.011 (0.122)	0.93
Systolic Pressure (mmHg)	0.054 (0.027)	**0.043**
Diastolic pressure (mmHg)	0.028 (0.017)	0.096
Waist (cm)	0.101 (0.098)	0.31
Insulin (µUI/ml)*	0.001 (0.005)	0.52
HOMA-IR*	0.001 (0.002)	0.62

Adjusted by sex and age.

*Values were logarithmically transformed.

*B* represents change in outcome per each additional copy of the risk (evening) allele.

Abbreviations: ME, morning-evening; MetS, Metabolic Syndrome; HDL-C, High Density Lipoprotein – Cholesterol; HOMA-IR, Homeostatic Model Assessment – Insulin Resistance.

### Chronotype and Behavioral lifestyle characteristics

Differences were observed between morning and evening chronotypes in several key lifestyle factors (Table 3).

**Table 3:** Lifestyle factors of ONTIME population.

	Morning Chronotype (Mean ± SEM) *n* = 1,110	Evening Chronotype (Mean ± SEM) *n* = 1,016	*P-value*	*P-trend*
***Dietary intake***				
Total energy (kcal/day)	1972.81 ± 23.83	1918.55 ± 24.68	0.121	0.935
Proteins (g)	83.01 ± 1.11	82.34 ± 1.15	0.677	0.935
Carbohydrates (g)	204.59 ± 3.07	193.78 ± 3.18	**0.017**	0.667
Fats (g)	93.79 ± 1.50	93.03 ± 1.54	0.729	0.487
***Food timing***				
Breakfast time (h)	8.34 ± 0.03	8.65 ± 0.035	**<0.001**	**<0.001**
Lunch time (h)	14.55 ± 0.02	14.59 ± 0.02	0.184	**0.002**
Dinner time (h)	21.08 ± 0.06	21.39 ± 0.67	**0.001**	**0.007**
Midpoint of intake (h)	14.80 ± 0.02	15.06 ± 0.02	**<0.001**	**<0.001**
***Eating behavior***				
Total Score	0.01 ± 0.25	1.93 ± 0.26	**<0.001**	**<0.001**
***Emotional eating***				
Total Score	11.85 ± 0.19	12.40 ± 0.19	**0.046**	**<0.001**
***Physical activity***				
IPAQ score	4282.60 ± 217.01	3229.77 ± 224.86	**0.001**	**<0.001**
Sitting duration (hours/day)	7.58 ± 0.11	8.15 ± 0.12	**<0.001**	**0.002**
***Sleep characteristics***				
Wake time, hours	7.25 ± 0.03	7.56 ± 0.03	**<0.001**	**<0.001**
Bed time, hours	23.73 ± 0.03	24.05 ± 0.04	**<0.001**	**<0.001**
Sleep duration, hours	7.53 ± 0.04	7.52 ± 0.04	0.835	0.424
***Smoking***				
Current smokers (%)	18	21	0.107**	
Number of cigarettes per day	10 ± 1	13 ± 1	**0.003**	**0.024**

Chronotype was dichotomized into “more morning” (Morning type) and “more evening” (Evening type) based on the median ME score of the total population (<53, more evening; ≥53, more morning).

Abbreviation: IPAQ, International Physical Activity Questionnaire.

Adjusted by sex, age, clinic site and number study.

*Values were logarithmically transformed.

**P-value was calculated using chi-square test.

*P*-value refers to association between morning and evening chronotype and exposures of interest. *P*-trend refers to the continuous association between the ME score and exposures of interest.

With respect to dietary intake, subjects with later chronotype had a lower carbohydrate intake, particularly related to the intake of cereals, a delayed breakfast, lunch and dinner, and a later midpoint of food intake (*P* < 0.05). Furthermore, evening chronotypes had a significantly higher eating behavior score than morning chronotypes (*P* < 0.001) suggesting more deleterious eating behaviors. Similar results were found for emotional eating score suggesting greater emotional influence on their eating behavior. After logistic regression analyses it was demonstrated that evening chronotypes had 1.3 times higher odds of stress-related eating, more difficulties in controlling the amount of food eaten (such as portion sizes, having second rounds, being prone to eat energy-rich foods) or to drink alcohol, compared to morning chronotypes. Similar findings were found for other detrimental eating behaviors and for emotional eating-related questions (Table 4).

**Table 4:** OR for Evening vs Morning chronotype in relation to eating behavior and emotional eating (*n* = 2,126).

**Eating behavior**	Evening vs Morning Chronotype		*P*
	OR	95% CI	
Are your portion sizes on the large side?	1.44	1.18, 1.77	**<** **0.001**
Do you take second servings?	1.27	1.04, 1.56	**0.019**
Are you prone to eat energy-rich (i.e., high-fat) foods?	1.44	1.16, 1.78	**0.001**
Do you drink alcohol?	1.52	1.25, 1.86	**<** **0.001**
Do you eat in places other than the kitchen or dining room?	1.32	1.04, 1.70	**0.023**
Do you eat while watching television?	1.23	0.99, 1.52	**0.065**
Do you eat directly from packets or containers?	1.31	1.06, 1.62	**0.013**
Are you prone to stress-related eating?	1.27	1.04, 1.55	**0.019**
Adequate cereals in the diet based on Mediterranean Diet definition (>218 g)*	0.75	0.60, 0.93	**0.009**
**Emotional eating**			
Do you crave specific foods?	1.20	0.99, 1.45	**0.063**
Do you have problems controlling the amount of certain types of food you eat?	1.31	1.08, 1.58	**0.006**
Do you feel less control over your diet when you are tired after work at night?	1.33	1.10, 1.60	**0.003**

ORs were calculated for combined groups of E-type compared with M-type.

Adjusted for sex, age, BMI, study number and clinic center.

*Computed from 24-hour dietary recall.

Other differences in lifestyle factors were observed between evening and morning chronotype. For physical activity, as assessed by IPAQ, evening chronotypes engaged in less physically activity and spent longer hours sitting per day (*P* < 0.05) compared to morning chronotypes. These associations remained significant after adjustment for BMI (*P* < 0.001 for both). In addition, evening chronotypes had later wake and bed times compared to morning chronotypes (*P* < 0.001), although no significant differences were observed for sleep duration. No significant association was found between current smoking status [smokers (n = 705) and nonsmokers (n = 2961)] and chronotype. However, among current smokers (19%), we found that subjects with late chronotype consumed more cigarettes *per day* than early chronotype (M ± SEM; later: 13 ± 1 vs early: 10 ± 1) (*P* = 0.003).

### Daily patterns of rest-activity rhythm and wrist temperature

**Figure 2:**
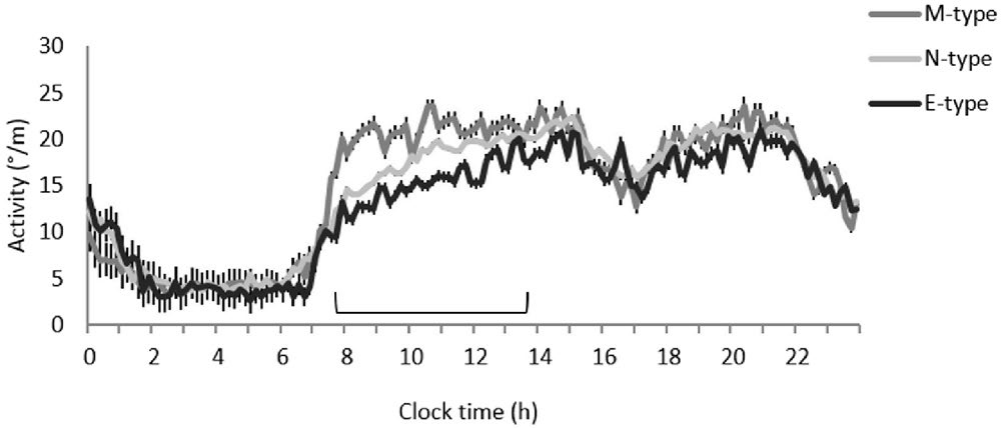
Daily mean waveform of actimetry recorded over an 8-day period in M-type (n = 15), I-type (n = 52) and E-type (n = 12) women. The section of the graph with significant differences (P < 0.05) between the different chronotype groups is highlighted in the figure.

Following 8 days of continuous monitoring of the rest-activity rhythm, we found significant associations between actimetry-derived parameters and chronotype: evening chronotypes had a delayed acrophase (Table 5). Furthermore, data consistently showed that morning chronotype were significantly more active during the morning and early afternoon (Fig. 2). Moreover, evening chronotypes started their physical activity later in the morning than the morning types.

**Table 5:** (n = 100) Associations between Circadian-related parameters and ME Score.

	**ME Score**		
	β	SEM	*P-value*
***Rest-activity rhythm***			
Acrophase, min	−0.051	0.012	**<0.001**
Time of 5-h LA, min	−0.041	0.011	**0.001**
Time of 10-h MA, min	−0.061	0.024	**0.013**
***Wrist temperature***			
PR, %	0.218	0.100	**0.031**

Abbreviations used: MEQ Score, Morningness-Eveningness Questionnarie. PR; Percentage of rhythmicity. Time of 5-h LA; Time of 5 hours of minimum activity. Time of 10-h MA; Time of 10 hours of maximum activity.

Adjusted by age, BMI, sleep hours and menopause status.

For wrist temperature, we observed that more evening chronotype was associated with lower percentage of rhythmicity (PR; *P* = 0.031; Table 5). Evening chronotypes had significantly lower interdaily stability (0.40 ± 0.03) than intermediate chronotypes (0.41 ± 0.02) and morning chronotypes (0.50 ± 0.03) (*P* = 0.046) and they had a trend towards a lower circadian function index (CFI) than the other chronotypes (Evening chronotype, 0.441 ± 0.010; Intermediate chronotype, 0.446 ± 0.006; Morning chronotype, 0.474 ± 0.012; *P* = 0.076).

Further discriminant function analysis including genetics and behavioral lifestyle factors demonstrated that eating patterns and sedentary behaviors such as sitting hours *per* day were able to reliably classify subjects into two chronotype groups (morning and evening) in 57% of the cases (*P* < 0.0001; for both eating patterns and sedentary habits) (Supplemental Table 2).

## Discussion

**Figure 3:**
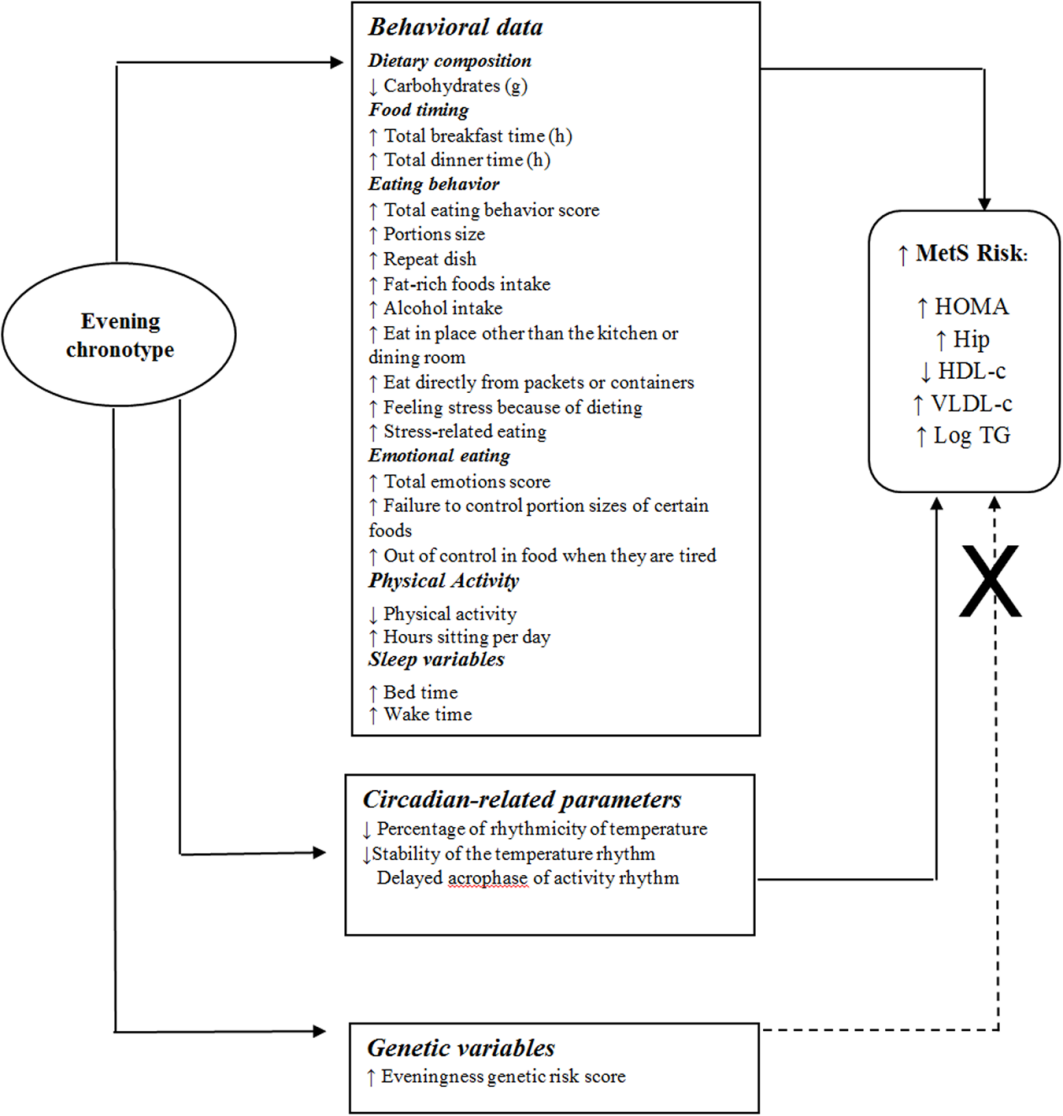
Potential mechanisms by which evening chronotype may predispose to MetS Risk.

In our population of 2,126 participants we observed that evening chronotype associates with adverse metabolic outcomes including higher BMI, greater insulin resistance and a higher total metabolic syndrome score, and further associates with several key lifestyle factors such as detrimental eating behaviors related to how, what and when they eat, lower physical activity and later sleep timing. Using a GRS comprised of recently identified chronotype-related genetic variants, we demonstrated a significant association between a higher evening GRS and evening chronotype, but did not observe an association of higher GRS with metabolic syndrome, which suggests that genetics are capturing chronotype but not the associated metabolic risk. To the best of our knowledge, this is the first study that examines the contribution of genetics, lifestyle factors, and circadian-related parameters in chronotype and related risk of metabolic syndrome (Fig. 3).

Consistent with earlier findings, we observed that evening chronotype was associated with a higher prevalence of obesity, metabolic syndrome and insulin resistance^20^. More evening chronotypes had a higher BMI, MetS Score and HOMA-IR as well as higher triglycerides and lower HDL compared to morning chronotypes, these results confirm previous data obtained in general populations^20,21^ and in type 2 diabetic subjects^22^.

Our GRS comprised of recently identified chronotype SNPs associated with self-reported chronotype. This is consistent with chronotype heritability determined from twin studies^23^. The genetic variants selected were discovered in large populations-based genome-wide association investigations of self-reported morning/evening preference including individuals of European ancestry from the UK Biobank^8,10^ and 23andMe^9^, and point to the biological contribution to inter-individual variability in chronotype, including variants related to the molecular clock machinery. The GRS that we have developed also captured chronotype in our population of European ancestry from Spain, despite differences in lifestyle, such as sleep hours, light exposure, activity and food timing (all synchronizers of the biological clock) compared to earlier investigations. Our finding suggests that the GRS derived from GWAS may be useful to capture the biological component of chronotype in different populations.

Since our GRS did not appear to relate to metabolic risk, we suspect that chronotype genetics may contribute to preferred evening chronotype, but is not a determinant of metabolic risk. This suggest that the associated risk between self-reported evening chronotype and metabolic risk may be modified by the behaviors we investigated, particularly physical activity, sitting hours per day, and eating behaviors.

With regards to the timing of food intake, we observed that evening chronotype associates with later intake of all three main meals. Similar trends of later intake have been consistently observed among evening chronotypes in other studies^21,24^. Emerging research in the field of nutrition has focused on meal timing (when) as a novel dimension of dietary intake in addition to meal composition (what) and eating behaviors (how). We have shown that eating late not only can decrease resting-energy expenditure and glucose tolerance, but also may blunt the daily cortisol rhythm and thermal effect of food^25^. Moreover, it was demonstrated that dietary and surgical weight-loss interventions are less effective in late eaters, although energy expenditure, calorie intake, and sleep duration are comparable to early eaters^26,27^. These metabolic alterations possibly contribute to the development of obesity and insulin resistance in late chronotypes.

On the other hand, eating behaviors associated with chronotype in the present study. Differences were observed in controlling the amount of food consumed, as evening chronotypes are more likely to have larger portion sizes, second rounds, and energy-rich foods. These obesity-related behaviors have also been associated with an increase in the methylation of several clock genes that characterize obese subjects^28,29^. Moreover, higher emotional eating score, observed among evening chronotypes, may indicate a greater role of emotions rather than endogenous hunger cues^19^. Whereas behaviors related to controlling the amount of food controlled would suggest higher energy intake among evening chronotype, we did not observe higher energy intake among evening chronotype based on the 24-hour recall. This seemingly contradictory finding may be related to the different tools used to assess food intake behaviors (questionnaire *vs* a single 24-hour recall) or may reflect food perception and attitudes rather than actual dietary intake. In addition, upon further investigation, we observed that the lower carbohydrate intake among evening chronotype was related to lower intake of healthful wholegrain cereals, a determinant of the Mediterranean Diet^30^.

The high prevalence of metabolic disorders in late chronotypes may in part be explained by physical inactivity and other unhealthy lifestyle habits. We found that subjects with evening chronotype were less physically active and spent longer hours sitting per day, both independent risk factors for metabolic disorders. Our results are in agreement with findings from other investigations in adults and adolescents that show that evening chronotype associates with lower leisure time physical activity and time spent in moderate-to-vigorous physical activity, and longer durations of sitting per day^17^. Previous findings observed that evening chronotypes had more screen time by 48 minutes and 27 minutes less moderate-to-vigorous physical activity per day relative to morning chronotypes, independent of sleep duration^31^. Our discriminant analysis indicates that eating patterns and sedentary behaviors were the two lifestyle factors that reliably characterized evening chronotypes.

Our objective measure of activity using 8 days of continuous monitoring of 24-h rest-activity rhythms supported these results, further indicating a lower activity level among evening chronotypes, particularly during earlier hours of the day between 8 AM and 2 PM. Furthermore, as expected, evening chronotypes had a delayed phase in activity, as ascertained by acrophase, time of L5 and time of M10 values. It has been reported that the timing of activity patterns from actigraphy correlates with the timing in melatonin rhythms^32^. Therefore, the observed delayed acrophase in evening chronotype may be due to delayed melatonin rhythms compared to early chronotypes. Future studies, measuring dim light melatonin onset are necessary to test this hypothesis.

Together with actigraphy, wrist temperature has been also proposed as a useful method to explore circadian rhythms and their potential association with aging, dementia^33,34^, and metabolic alterations^35^, although both are also influenced by social and environmental factors. Our results of wrist temperature show that evening chronotypes had a less robust (lower PR) and a less stable rhythm of wrist temperature and tended to have a weakened CFI. An altered pattern in wrist temperature with reduced PR has been previously associated with obesity and metabolic alterations^35,36^ and with increased levels of ghrelin (orexigenic hormone)^36^. The relatively low CFI in evening chronotypes, and the altered pattern in rest activity and wrist temperature daily rhythms could partly contribute to metabolic derangements in evening chronotypes^37^.

Some limitations should be noted in the present study. Our use of a single 24-hour dietary recall whereas sufficient to detect differences between morning and evening chronotypes, may be inadequate for capturing habitual dietary intake. Our cross-sectional findings limit our interpretation of the link between chronotype, metabolic alterations, and lifestyle factors and establishing causality. Longitudinal epidemiologic studies are required to elucidate directionality of these associations. However, in contrast to lifestyle factors, the current GRS could be used in Mendelian Randomization studies to establish causality due to random inheritance of genetic variants and lack of reverse causation in other populations^38^, opening a new window into understanding causality in epidemiologic studies. As our findings linking chronotype through behavioral lifestyle to metabolic syndrome pertain to a Mediterranean population of obese subjects, whether these findings are generalizable to other population of different ancestries and demographic is unclear.

In summary, first, we demonstrate the use of a chronotype GRS as a tool to capture the biological component of the inter-individual differences in chronotype, and second, we provide insight into modifiable lifestyle factors that may underlie the relationship between evening chronotype and metabolic alterations. Therefore, modifying behavior may attenuate metabolic risk of evening chronotypes. Specifically, limiting sedentary lifestyle, reducing detrimental eating behaviors particularly towards smaller portion sizes and selection of less energy-rich foods, and designing cognitive therapies to control emotional eating collectively may be effective strategies to reverse and prevent cardiometabolic chronic diseases among evening chronotypes. This knowledge may be applied to clinical practice and contribute to personalized therapies for the prevention of metabolic disorders.

## Materials and Methods

### Study population

A total of 2,126 overweight and obese subjects, 404 men and 1,722 women, (BMI: 31 ± 5 kg/m^2^; age: 40 ± 13) from the Obesity, Nutrigenetics, Timing, and Mediterranean (ONTIME) study were considered in the current study (clinicaltrials.gov: NCT02829619). Participants’ data were codified to guarantee anonymity. Participation was voluntary, subjected to informed consent. All procedures were in accordance with good clinical practice.

The registry and data collection procedures have been approved by the Committee of Research Ethics of the University of Murcia and it follows national regulations regarding personal data protection. Applicable institutional and governmental regulations concerning the ethical use of human volunteers were followed during this research. Lifestyle characteristics and blood measures were assessed at the same hour of the day for all participants at baseline. Genetics was also determined in 1,693 subjects. A subsample of 100 women was randomly selected for one-week of rest-activity rhythms monitoring and wrist temperature.

### Chronotype ascertainment

Chronotype was assessed using the Morningness-Eveningness (ME) questionnaire, a 19-item scale developed by Horne and Östberg, and an ME score was computed^39^. Question 19 of the ME questionnaire reads, “Which one of these types do you consider yourself to be?” with response options “Definitely a morning type”, “Rather more a morning type than an evening type”, “Rather more an evening type than a morning type”, or “Definitely an evening type”. The ME score was expressed continuously. We further dichotomized the score into “more evening” and “more morning” based on the median ME score of the total population (<53, more evening; ≥53, more morning) in order to facilitate interpretation and possible future clinical application.

### Anthropometric measurements and body composition

Subjects were weighed on a digital body weight scale to the nearest 0.1 kg while barefoot and wearing light clothing. Subject’s height was assessed by a Harpenden digital stadiometer (with a rank of 0.7–2.05). Participants were instructed to stand in a relaxed upright position with their head oriented in the Frankfurt plane. BMI was calculated as weight (kg)/height^2^ (m). Waist circumference was measured at the level of the umbilicus. Body fat composition was ascertained with bioelectrical impedance, using the TANITA TBF-300 equipment (Tanita Corporation of America, Arlington Heights, IL, USA).

### Metabolic syndrome components and insulin resistance

Fasting glucose was determined in serum with the glucose oxidase method^40^. Plasma concentrations of triglycerides and high-density lipoprotein (HDL) cholesterol were determined with commercial kits (Roche Diagnostics GmbH, Mannheim, Germany). Arterial pressure was measured with a mercury sphygmomanometer. MetS score was computed for each subject per the International Diabetes Federation criteria by summing each of the MetS components (waist circumference, fasting glucose, triglycerides, HDL-c, and systolic and diastolic blood pressures)^41^.

Fasting insulin was determined through a solid-phase, two-site chemiluminescent immunometric assay (IMMULITE 2000 Insulin). Homeostasis model assessment of insulin resistance (HOMA-IR; fasting glucose × fasting insulin/22.5) was used to assess insulin resistance (IR).

### Lifestyle factors

#### Dietary composition and food timing

Dietary intake was self-reported for breakfast, lunch, and dinner using a single 24-hour recall to evaluate habitual dietary intake. Total energy intake and macronutrient composition were analyzed with the nutritional evaluation software program Grunumur 2.0^42^, based on Spanish food composition tables^43,44^. Food timing (clock times) was also self-reported for each meal. Midpoint of intake was ascertained by calculating the midpoint between breakfast and dinner times (first and last eating episode).

#### Eating Behavior

An Eating Behavior Score was computed for each participant based on responses to the Barriers to Weight-Loss checklist^45,46^. The 29-item checklist consists of 7 sections as follows: meal recording weight control and weekly interviews; eating habits; portion size; food and drink choices; way of eating; and other obstacles to weight-loss^45^.

#### Emotional Eating

The Emotional Eating Questionnaire (EEQ) was used to assess emotional eating behavior. The 10-item questionnaire has been created to assess the extent of the influence of emotions on eating behavior^47^. An EEQ score was computed for each subject, then dichotomized into emotional and non-emotional eaters based on the median emotional score of the total population (<12, non-emotional; ≥12, emotional).

#### Physical activity and sitting duration

The International Physical Activity Questionnaire (IPAQ) was administered with assistance from a nutritionist to assess physical activity (PA) during the 7-days prior to enrollment^48^. The IPAQ has been validated internationally and in a Spanish population, in which good correlation with accelerometer data were obtained^48,49^. A total PA score reflecting intensity and time was calculated in MET (metabolic equivalent of task) minutes per week for the four IPAQ domains combined. Subjects with METs/week <600 METs/week were classified as sedentary. Sitting duration in hours/day was further assessed by the question: “How many hours per day do you usually spend sitting?”

#### Sleep characteristics

Bed and wake times were also self-reported. Habitual sleep duration in hours was calculated using the difference between bed and wake times.

### Circadian-related parameters

The rest-activity rhythm was assessed over the same 8 days using a HOBO Pendant G Acceleration Data Logger UA-004- 64 (Onset Computer, Bourne, MA) placed on the non-dominant arm with a sports band, placing the x axis parallel to the humorous bone. The sensor was programmed to record data every 30 seconds. Data were extracted using the software provided by the manufacturer (HOBOware 2.2)^50^.

Wrist Temperature was assessed continuously over 8 days using a temperature sensor (Thermochron iButton DS1921H; Dallas Maxim, WI) with a sensitivity of 0.1258 °C and programmed to sample every 10 minutes. The sensor was attached to a double-sided cotton sport wristband, and the sensor surface was placed over the inside of the wrist on the radial artery of the non-dominant hand, as previously described by Sarabia *et al*.^37^. Data were extracted using iButton Viewer v. 3.22 (Dallas Semiconductor MAXIM software, provided by the manufacturer). Data were recorded from November to May, with environmental temperatures ranging between 16.2 °C and 21.4 °C (data obtained from the Centre for Statistics of Murcia), to limit the influence of extreme environmental temperatures on wrist temperature.

The reliability of wrist temperature and rest-activity rhythm parameters has been previously validated with polysomnography (PSG)^51^. The following parameters were derived from actimetry and wrist temperature.

### DNA isolation and genotyping and calculation of a genetic risk score (GRS)

A set of 18 single nucleotide polymorphisms (SNPs) was derived from 3 recent chronotype genome-wide association (GWA) studies (Supplemental Table 1). DNA was isolated from blood samples using standard procedures (Qiagen, Valencia, CA, USA). Genotyping of chronotype SNPs were performed using a TaqMan assay with allele-specific probes on the ABI Prism 7900HT Sequence Detection System (Applied Biosystems, Foster City, CA, USA).

A weighted GRS was generated for the chronotype SNPs. Among the selected 18 SNPs, 3 SNPs had minor allele frequencies <0.01 [rs141175086, rs11895698, and rs148750727] and were subsequently excluded from this analysis. The remaining 15 common SNPs had genotype frequencies consistent with Hardy–Weinberg equilibrium (*P* > 1 × 10^−6^) and passed quality control. Individual participant scores were created by summing the number of risk alleles at each genetic variant weighted by the respective allelic effect sizes on risk of evening chronotype from the genome-wide association by Lane *et al*.^7^ for chronotype as assessed by Question 19 of the ME questionnaire. GRS values were normally distributed across the population. Furthermore, individual SNP association analyses were conducted using an additive genetic model. GRS association analysis was performed in participants with complete genotyped data, whereas individual SNP association analyses were performed in participants with genotype data for that particular SNP of interest.

### Statistical analysis

Non-normally distributed variables, triglyceride levels, MetS, and HOMA-IR, were logarithmically transformed. We performed analysis of covariance to analyze differences between morning and evening chronotypes. Linear regression was also used to test for associations between chronotype ME score (continuous) and outcomes. Moreover, we fitted logistic regression models to estimate the odds ratios (ORs) and 95% CIs of eating behavior and emotional eating and chronotype. We adjusted all analyses for sex, age, clinic site, and study number. Similar linear regression analyses were performed for association between chronotype ME score and chronotype GRS, or individual SNPs, and adjusted for age and sex.

A stepwise discriminant analysis was applied to the set of variables that significantly differed in univariate analysis between chronotypes. The final analysis indicated the variables with the greatest contribution in discriminating between the two groups, and finally, the discriminant model was validated by checking the percentage of group cases correctly classified after cross-tabulation of actual and predicted group membership provided by the discriminant function.

To characterize wrist temperature and activity rhythms, we calculated several rhythmic parameters (see appendix 1) with parametric and nonparametric methods by using an integrated package for temporal series analysis Circadianware (Chronobiology Laboratory, University of Murcia, 2010). To assess for a) associations between the individual chronotype and the circadian-related parameters obtained, we used logistic regression analyses for chronotype as continuous variable, and analysis of covariance for assessing differences between three chronotype categories (morning, intermediate, and evening). All analyses were adjusted for age, BMI, menopause status and sleep duration. Differences in actimetry pattern among morning, intermediate, or evening chronotypes were determined by analysis of repeated measurements during the 8-day period at every 30-second interval.

Statistical analyses were conducted using SPSS 20.0 software (SPSS, IBM, Madrid, Spain). A two-tailed P-value of <0.05 was considered as statistically significant.

## Electronic supplementary material

Supplementary Information
