# Supplementary material for: Modifiable lifestyle behaviors, but not a genetic risk score, associate with metabolic syndrome in evening chronotypes
Source: Sci Rep. 2018 Jan 17;8:945. doi: 10.1038/s41598-017-18268-z (PMC5772646; doi:10.1038/s41598-017-18268-z)
Supplement: Supplementary file 1 — Supplementary Information [file 41598_2017_18268_MOESM1_ESM.pdf]

Title: Modifiable lifestyle behaviors, but not a genetic risk score, associate with metabolic syndrome in evening chronotypes.

Authors: Beatriz Vera[1]**, Hassan S Dashti[2]**, Purificación Gómez-Abellán[1], Antonio M. Hernández-Martínez[3], Alberto Esteban[4], Frank AJL Scheer[5,6], Richa Saxena[2^]*, Marta Garaulet[1^]*.

Appendix 1.

Term	Definition
Acrophase	Timing of the maximum value of the cosine function
Circadian Function Index (CFI)	A numerical index that determines the circadian robustness, based on three circadian parameters: Interdaily stability (IS), Intraday variability (IV) and Relative Amplitude (RA). CFI oscillates between 0 (absence of circadian rhythmicity) and 1 (a robust circadian rhythm).
Interdaily Stability (IS)	The similarity of the 24 h pattern over days; varied between 0 for Gaussian noise and 1 for perfect stability, where the pattern repeated itself exactly day after day.
Intraday Variability	Characterizes the rhythm fragmentation
M5	The average of the 5 consecutive hours of minimum activity values
M10	Average 10 consecutive hours of maximum activity value
Percentage of rhythmicity (PR)	Percentage of variance of data explained by the sinusoidal function. Higher values of this parameter mean a more sinusoidal curve.
TM5	Timing of M5
TM10	Timing of M10

Supplemental table 1. General characteristics of population with Genetic Risk Score

n=1,693	**Mean**	**SD**
Age (y)	40.53	12.42
%Women, n (%)	1363 (80.65)	-
BMI (kg/m^2)	31.26	5.41
Body fat (%)	37.17	6.71
Metabolic syndrome and insulin resistance		
Waist (cm)	102.83	14.89
Fasting glucose (mg/dl)	85.15	15.03
Triglycerides (mg/dl)	102.00	51.90
HDL-c (mg/dl)	56.43	14.89
Systolic Pressure (mmHg)	11.67	1.49
Diastolic pressure (mmHg)	7.22	1.00
MetS Score	2.12	1.17
Insulin (µUI/ml)	7.27	6.52
HOMA-IR	1.59	1.60

Abbreviations: BMI; Body mass index. MetS; Metabolic Syndrome. HDL-c; High Density Lipoprotein. VLDL-c; Very Low Density Lipoprotein-Cholesterol. HOMA-IR; Homeostatic Model Assessment-Insulin Resistance

Supplemental table 2. Cross-validation of discriminant function of chronotype.

Classification percent as			
**Actual group**	**Evening**	**Morning**	**Total N**
Evening	56 (n= 441)	44 (n= 350)	791
Morning	42 (n= 370)	58 (n= 511)	881

Overall classification accuracy=**57%**
